# Demonstration of active neutron interrogation of special nuclear materials using a high-intensity short-pulse-laser-driven neutron source

**DOI:** 10.1038/s41598-024-82641-y

**Published:** 2025-01-03

**Authors:** A. Favalli, D. C. Henzlova, S. Croft, O. Deppert, K. Falk, J. C. Fernandez, D. C. Gautier, N. Guler, C. E. Hamilton, K. D. Ianakiev, M. Iliev, R. P. Johnson, A. Kleinschmidt, M. Roth, T. N. Shimada, M. Swinhoe, T. N. Taddeucci

**Affiliations:** 1 Los Alamos National Laboratory, Los Alamos, NM 87544 USA; 2 Institut für Kernphysik, Technische Universität Darmstadt, 64289 Darmstadt, Germany; 3 Oak Ridge National Laboratory, Oak Ridge, TN 37831 USA; 4 Lancaster University, Bailrigg, Lancaster, UK; 5 Institute of Radiation Physics, Helmholtz-Zentrum Dresden-Rossendorf, Bautzner Landstr. 400, 01328 Dresden, Germany; 6 Technische Universität Dresden, 01062 Dresden, Germany; 7 Institute of Physics, Czech Academy of Sciences, Prague, 182 21 Czech Republic; 8 Spectral Sciences, Burlington, MA 01803 USA

**Keywords:** Engineering, Physics

## Abstract

Detecting shielded special nuclear material, such as nuclear explosives, is a difficult challenge pursued by non-proliferation, anti-terrorism, and nuclear security programs worldwide. Interrogation with intense fast-neutron pulses is a promising method to characterize concealed nuclear material rapidly but is limited by suitable source availability and proven instrumentation. In this study we have pioneered a demonstration of such an interrogation method using a high-intensity, short-pulse, laser-driven neutron source that offers potential benefits compared to conventional neutron sources. The measurement results reported here represent the first experimental demonstration of this interrogation approach on enriched uranium items and demonstrate the feasibility of a precise measurement using realistic nuclear materials, representative of field scenarios, even with just a single laser-driven neutron pulse. Bright pulsed sources can overcome the nuisance background of items with strong internal neutron sources, improving analytical power, while single-shot assay is attractive in high-throughput situations where time is at a premium. The science and technology of this type of neutron production is developing rapidly, and we anticipate that practical mobile interrogation systems will become available based on the detection concepts demonstrated here to meet the growing measurement needs.

**Supplementary Information:**

The online version contains supplementary material available at 10.1038/s41598-024-82641-y.

## Introduction

A short-pulse laser-driven neutron source (LDNS) offers a compact, bright, and relatively inexpensive way of producing high-intensity neutron-beam pulses that can be used for a variety of basic and applied scientific needs^1–27^. LDNSs typically operate by directing an intense ion-beam of protons or deuterons driven by a high energy, high-intensity laser into a suitable material acting as a fast (~ MeV) neutron converter via a suitable nuclear reaction, the so-called pitcher-catcher configuration. Since the ion-beam pulse is very short in duration (~ 1 ps at birth), placing the converter close to the laser target (~ 1 cm) yields a fast neutron burst (~ 1 ns). The kinematics of the nuclear reactions of the ion-beam in the converter gives predominantly forward directionality (~ 1 sr) to the neutron source. Using the Trident laser facility at Los Alamos National Laboratory (LANL), an advancement in the yield and efficiency (and therefore fluence and intensity) of laser-driven neutron-beam sources was demonstrated^4,15^ by driving deuteron ion beams in the relativistic transparency regime of laser-plasmas (the Breakout Afterburner (BOA) ion-acceleration mechanism)^13,28^. At the Trident laser facility, a sub-ps laser beam was focused to a peak intensity of 10^21^ W/cm^2^ on an ultra-thin (sub-micron) deuterated plastic foil target, to drive a high-energy deuteron ion beam, which was directed onto a beryllium converter disk placed behind the foil to produce a neutron beam via nuclear breakup reactions. This neutron source features both high intensity and predominantly forward directionality, both of which are operationally useful. Yields of > 10^10^ fast neutrons per sr per shot, peaked in the forward direction, with extremely short neutron pulse duration (~ 1 ns) can be routinely produced^4,5,17,20,21^.

A principal motivation for such a source was the capability to perform nondestructive assay (NDA) of special nuclear material (SNM) for nuclear material accountancy, nuclear safeguards, and national security applications in entirely new ways that are enabled by the unprecedented fast-neutron intensity in a single brief fast-neutron pulse^5^.

The introduction of such sources promises to transform many research and technological areas. The extensive review of Diven^29^ outlined the unique potential of the intense neutron flux from nuclear explosions to survey neutron cross sections and make unique measurements. Specifically, he describes the salient features, advantages and limitations of intense short-duration single-pulse ‘white’ neutron sources for time-resolved studies. LDNS sources allow for a reimagining of these possibilities on a more manageable, deployable, and feasible scale, as well as other traditional pulsed neutron generator application areas, such as those built around TRIGA burst-reactors, and large-scale accelerators^30,31^. To realize the potential of pulsed laser sources, understanding and controlling the beam characteristics is critical, as is mastery of how to adapt and optimize nuclear measurement instruments and methods to specific real-world problems such as the assay of special nuclear materials.

The detection, characterization, and quantification of nuclear materials is an evolving technological challenge being advanced by, for example, non-proliferation and anti-terrorism programs^32^. The detection of shielded special nuclear materials, including nuclear explosives^33^, to assist with interdiction of trafficking is a major concern, as too is the ability to perform materials analysis and energy-resolved neutron radiography^21,26,27^. In this context, active interrogation is particularly important for the detection of uranium as the passive emissions are relatively weak.

LDNS technology offers important advantages over existing neutron sources such as deuteron-tritium (DT) neutron generators^34^ or sealed radionuclide neutron sources (typically ^252^Cf spontaneous fission or AmLi (α,n) neutron sources), which have been the main sources available in the past for interrogation applications. These advantages are based on the ability to deliver in a single shot a relatively high yield of fast neutrons in a short burst over a relatively small solid angle, i.e., an intense, directed or ‘beamed’ neutron burst. Moreover, the technology exists for the relevant high-energy, high-power lasers to run at 10 Hz, allowing the possibility of accumulating yield rapidly to buildup information to meet a given data quality objective (see Discussion section, “Further work towards a production system”). The directionality of the neutron source provides an advantage in personnel safety and material activation concerns relative to isotropic sources with the same assay flux because of the simpler shielding considerations and higher useful neutron fraction. High intensity enables a high signal-to-noise ratio measurement in difficult situations such as the high background neutron emission rate from irradiated nuclear fuel. High yield enables short assay times, which translates to a high item throughput, which can be important in industrial settings. In addition, application-specific tailoring of the interrogating neutron energy spectrum and the emission angular distribution is possible and advantageous. For example, tailoring the neutron energy to lie in the range of a few MeV up to several tens of MeV allows relatively deep penetration into heavy (high Z) shielding materials, which poses an extreme challenge for γ-ray based interrogation technology. While high-power, high-energy lasers are not physically small, the technology exists to make them moveable for flexibility in the deployment of this high-value asset. The laser beam itself can be readily transported over relatively long distances (~ 10–100 m) into the vacuum chamber containing the laser-target and neutron converter. In a production (rather than research) environment, that chamber can be small with minimal footprint in the interrogation bay, providing flexibility and minimizing cost and complexity.

Active neutron interrogation of nuclear materials involves measurements of neutron induced signatures to identify/assay SNM during and after an interrogation with an external neutron pulse^35,36^. The concepts and theoretical foundation of methods for such interrogation have been long established^36^ but over the past few decades, practical realization has been made easier by the availability of powerful yet compact multi-parameter data acquisition chains and high-speed large-memory computers for experimental simulation and data analysis. A limiting factor is the availability of compact, reliable, bright neutron sources with favorable characteristics (pulse width, intensity, energy, and angular distribution), which has motivated the interest and vigorous research on laser-based neutron sources.

The neutron-induced signatures of interest include prompt and delayed fission neutrons emitted by the assayed item. The prompt and delayed components are defined here as corresponding to < 1ms following the interrogating neutron pulse for the former and > 1ms for the latter. The definition of prompt and delayed component timing varies in the literature. The one used in this paper is selected to separate any effects of the interrogating neutron pulse from the delayed neutron emission signal. This includes detector deadtime following the neutron pulse, room return of scattered near-thermal neutrons, and additional effects, all further discussed in the paper. Two active neutron interrogation techniques employed in current nuclear safeguards applications include differential die-away assay (DDA)^35^ and delayed neutron assay (DN)^37^, designed to exploit the neutron signatures in the prompt and delayed time intervals, respectively. Delayed neutrons (emitted isotropically following β-decay, hence “β-delayed”) represent a particularly attractive signature to detect SNM, since very few other processes besides nuclear fission yield neutrons on similar timescales giving the method high analytical specificity. Although the delayed neutron yield is only a few percent of the prompt yield, the time delay allows the neutron-signal from the interrogation neutron pulse to dissipate before the delayed neutrons are counted, which greatly improves the signal-to-noise.

Active neutron interrogation has been studied extensively and is implemented widely, with a primary focus so far being the measurement of low amounts of SNM in radioactive waste^38^. Generally, the interrogation is performed via pulsing a D-T neutron generator, with a frequency typically around 100 Hz and neutron intensities of the order of ~ 10^8^ n/s. The prompt and delayed fission neutrons are then detected in ^3^He-filled proportional counters^34–37^.

**Fig. 1:**
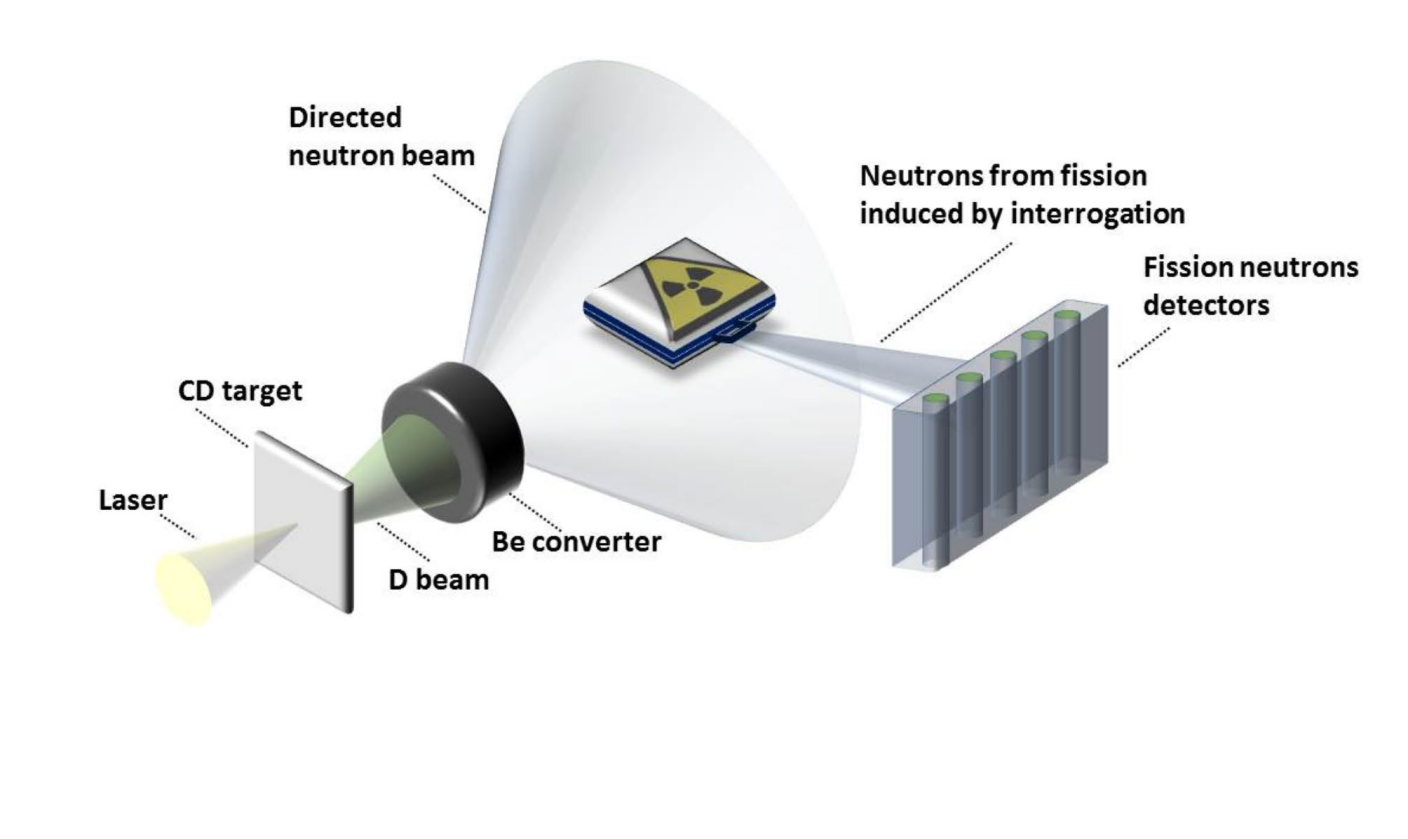
The concept of active interrogation using a laser-driven neutron source is presented. The objects being interrogated can vary in size, ranging from small items like suitcases to large objects such as freight containers.

The concept of active interrogation using a laser-driven neutron source is presented in Fig. 1. A laser interacts with a deuterated polystyrene (CD) target and produces deuterons (D beam), subsequently converted into a neutron beam in a Be puck. The neutron beam is then used for active interrogation of a concealed nuclear material object (here shown as a suitcase). The detection of induced fission neutrons is a signature of the presence of nuclear material and is used to assay the nuclear material in the object.

Dedicated experiments were conducted at LANL to assess a short-pulse LDNS for active interrogation of uranium materials. Exploration by direct experiment is presently necessary because a comprehensive end-to-end modeling tool from the laser plasma to the neutron output does not exist yet. Moreover, the impact of the unique radiation environment created by this novel source on the measurements on which a successful active interrogation strategy depends could not be confidently ascertained a priori. Experiments are, therefore, essential in both the development of active interrogation concepts and the associated simulation tools. Pursuant to that end, the experimental results obtained for enriched uranium using the β-delayed neutron counting technique are presented and discussed here. These measurements have provided the first-of-a-kind experimental demonstration of active interrogation using a high-intensity short-pulse laser-driven neutron source and demonstrate the feasibility of interrogation using a single laser-driven neutron pulse.

## Results

**Fig. 2:**
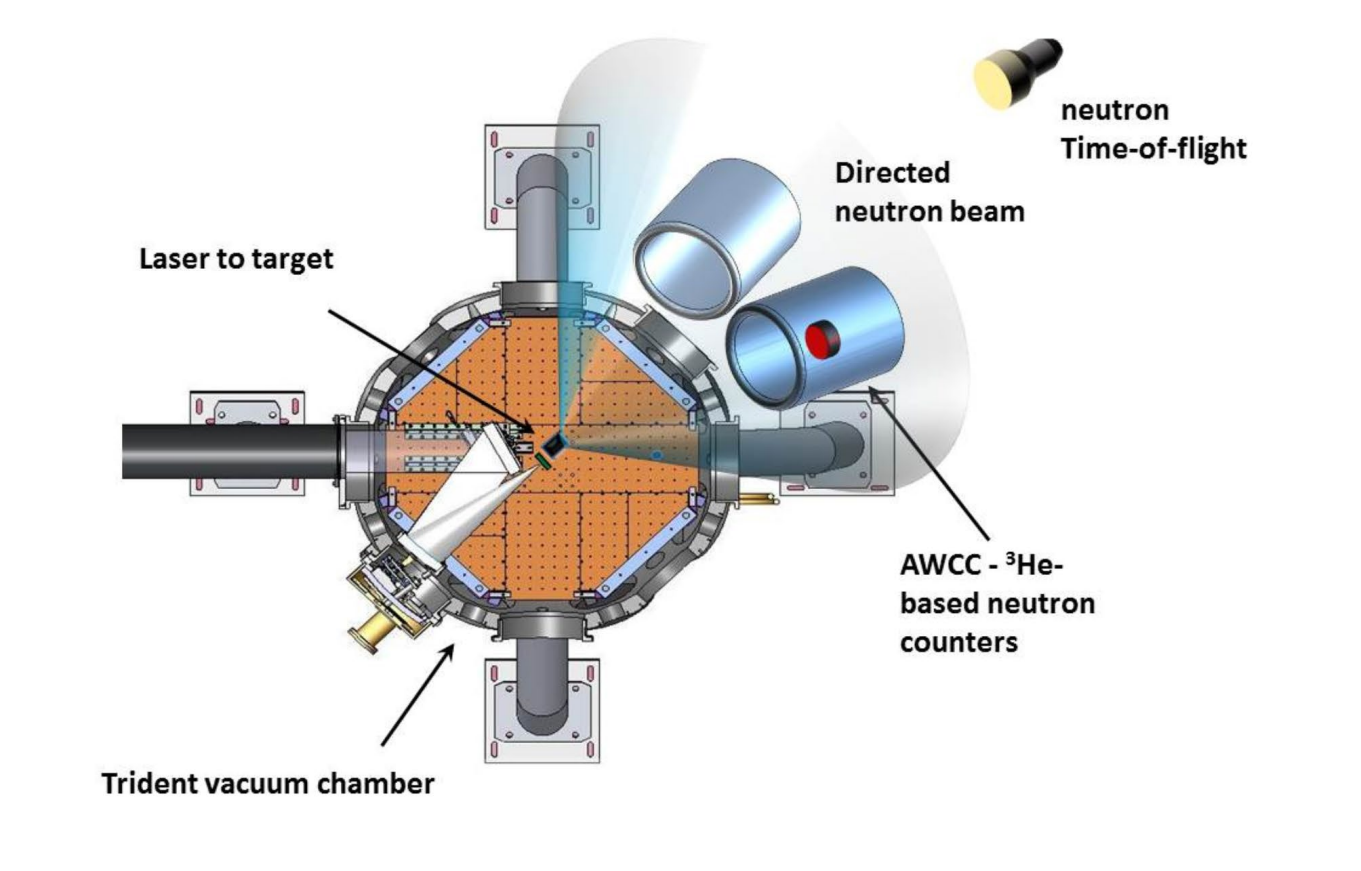
Set-up of the experiment (detector sizes and placement not to scale). The red item inside one of the ^3^He-based AWCC neutron detectors is a sample of special nuclear material. AWCC counters were positioned on the equator with their open end facing towards the neutron converter, closely straddling the central beam axis at an angle of ~ 10 degrees from that axis.

The experiments were carried out at the 200 TW Trident laser facility at LANL in the setup shown in Fig. 2.

The evacuated target chamber (20 mm thick stainless steel, 1 m in diameter) is equipped with Al flanges for equipment insertion and user access. It housed the laser turning mirror and focusing optics for the 9-inch (~ 229 mm) diameter laser beam. The chamber also housed the assembly for target support and positioning, the laser target, and the ion-to-neutron converter, of ~ 100 mm dimension overall. The laser focusing optic used in the present experiment was an f/1.5 off-axis parabolic mirror with a high-reflectivity Hf-coating for the 1.053 μm laser wavelength (note an f/3 arrangement is shown in Fig. 2 as various configurations were studied during the program of work). The typical 80 J high-contrast^39^ 0.6 ps laser pulses (FWHM) reached a peak vacuum intensity of ≈10^21^ W/cm^2^. The targets for this run were spin-coated polystyrene (CD) nanofoils 1 g/cm^3^ with thickness in the range of 0.6–0.75 μm, in order to access the Break-out Afterburner (BOA) laser-driven acceleration mechanism^39^ for the D-beam of interest in these experiments. A key to realizing this ion-acceleration regime on Trident was its high-contrast laser front end^39^, because without sufficiently high laser-pulse contrast, the pre-pulse and main-pulse pedestal would destroy such thin targets. The repetition rate in these experiments was approximately one shot about every 90 min. This rate was solely dictated by proper cooldown of the laser-amplifier glass in the last amplifier stage, which is flash-lamp pumped in this decades-old design.

The resulting high-energy D beam is directed at the ion-to-neutron converter. The converter consisted of a stack of Be cylinders 50 mm in diameter totaling 6 mm to 15 mm in thickness with their axes aligned along the laser-propagation direction (i.e., the ion-beam cone axis). The laser-facing end of the cylinder stack was located 38 mm downstream from the laser target and protected from the plasma and the transmitted light by Kapton tape to avoid damage and Be dispersal in the chamber.

The neutron diagnostic equipment, placed outside the vacuum target chamber, included one neutron time-of-flight (nTOF) detector system to measure the energy distribution of the laser-driven fast neutron production and two existing high-efficiency thermal-neutron well-counters (Active Well Coincidence Counters [AWCCs]) to measure the neutron emission from the SNM items. In this proof-of-principle experiment, rather than develop and qualify custom delayed-neutron detectors, as would be justifiable in a dedicated interrogation system, we used existing hardware for convenience. Indeed, AWCCs of this type are routinely used by the international nuclear safeguards and security communities to assay U and Pu by other means^40,41^. The AWCCs have a hollow cylindrical cavity where the interrogated sample may be placed. To unambiguously demonstrate the β-decay-delayed neutron signature following fission, the experimental campaign involved a comparative evaluation, where two identical detectors were used; one contained the interrogated sample (master counter), while the other remained empty and served as a reference (reference counter). Each of the AWCCs is equipped with 42 4-atm ^3^He-filled proportional tubes, each 25.4 mm in outer diameter, embedded in high-density polyethylene (HDPE). The tubes are arranged in two concentric rings surrounding the central well. In their standard configuration, each AWCC is equipped with top and bottom HDPE inserts that are used to minimize neutron leakage from the sample well. During the current experimental campaign, the detectors were oriented sideways (i.e., with their ‘top’ opening facing the laser target) to fully expose the interrogated material to the laser-produced neutron beam. In this arrangement, the top and bottom HDPE inserts were removed to allow for maximum penetration, even for the low-energy neutron beam component. To ensure identical measurement conditions for both detectors during the interrogation measurements, both detectors were positioned symmetrically relative to the axis of forward directed neutron beam, with the respective detector axes pointing toward the source (as shown in Fig. 2). Each AWCC is also equipped with a thin (~ 1 mm) Cd liner in the cavity, which blocks the contribution of slow (energies below about 0.4 eV) neutrons returning into the detector cavity after moderation in HDPE. Otherwise, such moderated fast fission neutrons from the sample returning to the cavity could induce additional fission in the sample resulting in an additional delayed-neutron signal. Thus, the AWCC has two operating modes: ‘fast mode’ (interrogation with fast neutrons) with the Cd liner in place and ‘thermal mode’ (interrogation with mainly thermal neutrons) with the Cd liner removed. The former case is suitable for interrogation of large quantities of U (0.1–20 kg). In the latter case, the sensitivity of the counter is greatly enhanced (due to the large thermal ^235^U fission cross section) that makes the counter more suitable for interrogation of small or low-enriched uranium materials (0–100 g of ^235^U). Both modes were investigated in the current experimental campaign.

Three ~ 900 g uranium samples with enrichments of 12%, 38%, and 66% of ^235^U were used. Both of the AWCCs used in the experiment were thoroughly characterized prior to the laser-driven interrogation measurements as explained in the Methods section.

In the following, firstly, we provide the characterization of the laser-driven neutron source (the interrogation source), and secondly, we report on the demonstration of the active interrogation of SNM.

### Neutron production

**Fig. 3:**
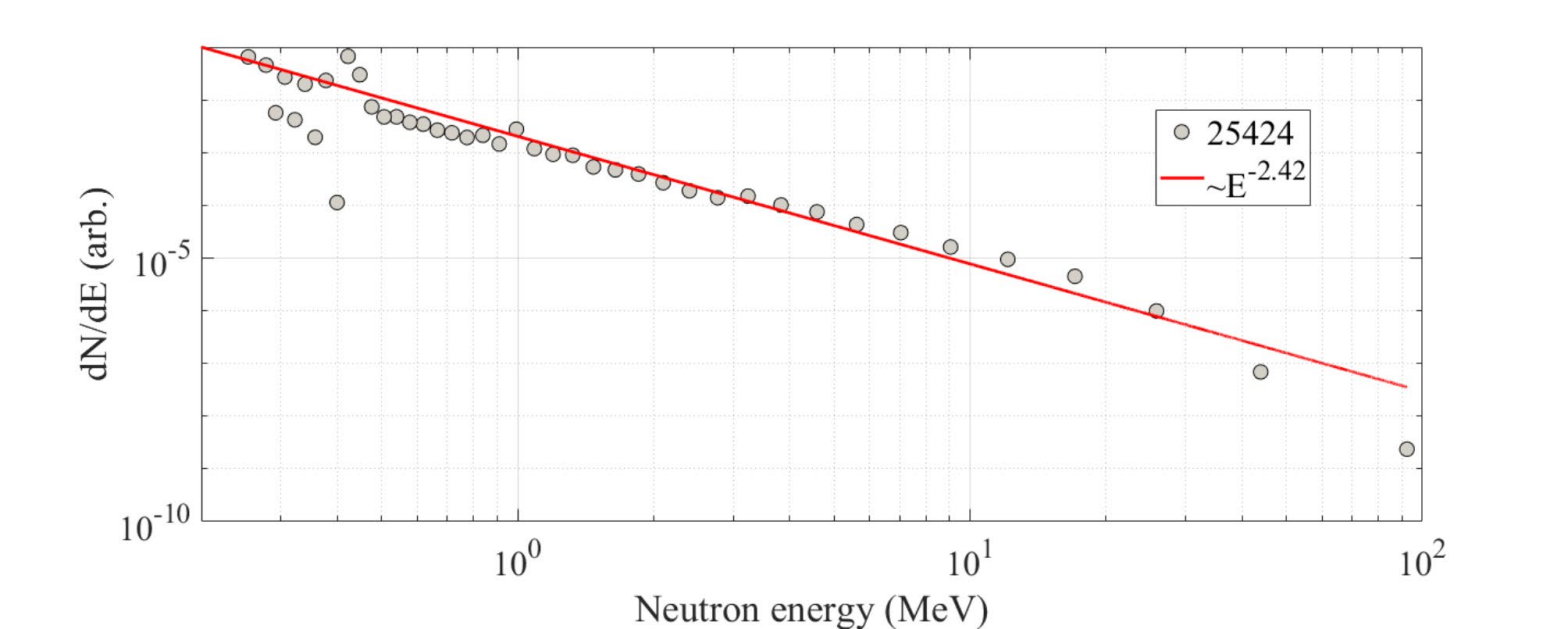
Neutron energy spectrum as extracted by the measurements of neutron-time-of-flight of the 36.7 ± 0.6 mrem shot (see Table 1, shot number 25424) with 700 nm CD target thickness and 6 mm Be thickness, 80.5 J on target. The energy spectrum approximately exhibits a power-law distribution, $\:\sim{E}^{-2.42}$, where E is the kinetic energy of the neutrons in MeV.

A neutron time-of-flight (nTOF) detector system was located outside the building at 6.2 m from the neutron beryllium converter. That location made it possible to measure the high energy component of the neutron spectrum with good resolution (due to the distance), to separate in time the signal from the prompt “gamma flash” (the burst of X-rays from the laser-plasma interaction) to allow recovery of the photomultiplier tube detector and electronics, and to avoid the problem with neutron signals in the detector coming from scattering neutrons in the experimental room.

The absolute neutron yield per pulse was measured for each shot using bubble detectors positioned around the chamber, including the target chamber exit flange directly on axis in the forward direction of the neutron beam. The bubble detectors are sensitive to neutrons with energy from a few 100’s of keV up to 100’s of MeV^11^. The determination of the absolute neutron yield per sr from the bubble detector and TOF diagnostics is explained in the Methods section.

Figure 3 shows the derived neutron energy spectrum (see the Methods section) for laser shot 25,424, where a 700 nm thick CD target, and 6 mm Be converter were used, with 80.5 J of laser energy on target, and a neutron dose of 36.7 ± 0.6 mrem (1-σ) was produced (determined by averaging bubble detection readings from bubble detectors with low and high sensitivity, see Methods, located at the exit flange). The energy spectrum resembles a power law, $\:dN/dE\sim{E}^{-2.42}$ with E being the kinetic energy of neutrons in MeV, up to about 3 MeV, with a residual of very fast neutrons with energies extending up to ~ 50–100 MeV. The energy spectrum presented is typical of our shots^42^. In the same campaign (for active interrogation) we achieved reproducibility of shots at the same neutron dose level (shot 25434: 82.2 J 600 nm CD target, 9 mm Be, 36.9±0.6 mrem; shot 25451, 83.2 J, 650 nm Cd target, 15 mm Be, 35.5±1.2 mrem; shot 25456: 77.3 J, 700 nm CD target, 15 mm Be 35.9±1.0 mrem).

The neutron dose of 36.9 mrem at the exit flange corresponds to a neutron yield of (2.1±0.3) ×10^10^ neutrons/sr in the neutron energy interval 0.2 MeV to 92.4 MeV, once the neutron spectrum and the dose-to-yield conversion curve for the bubble detectors are used (see Methods). The uncertainty includes the estimated contributions from the bubble detector reading, the spectrum deconvolution process, and the interpolation of the bubble detector dose-to-yield conversion curve. The yields for the shots reported above are all above 2.0 × 10^10^ neutrons/sr. The consistent and reproducible high-intensity neutron yield per shot reported here is the result of incremental improvements in laser operations and target instrumentation. These improvements have optimized beam readjustment, and enhanced target alignment, leading to improved performance in neutron generation^43^. The ability to extract the actual neutron energy spectrum and improvements in target thickness and Be converter radius design have also contributed to the reported performance.

### Detection of nuclear material by active interrogation

In the present experiments, the two AWCC neutron detectors were positioned in a direct line of sight of the forward portion of the neutron beam (see Figs. 1 and 2). One contained the interrogated material in its central well, while the other served as an empty reference or blank for comparison. The campaign comprised a series of individual laser-driven neutron pulses, each a few ns in duration, separated by approximately 90 min required for replacement of the laser-target, cool-down of the laser, re-alignment of the laser beam (if needed), and pumping the vacuum chamber. Data from the AWCC detectors were acquired in the form of timestamps corresponding to every neutron detection event using a PTR-32 list mode data acquisition unit^5^. The PTR-32 is a multi-input device that records neutron detection times (leading edge of the logic pulses from the charge-amplifier-discriminator circuit attached to the ^3^He proportional counters) with a resolution between two subsequent detection events of 10 ns. This data format allows for direct investigation of the temporal behavior of detected neutrons following an interrogating neutron pulse and enables a direct visualization of the delayed neutron signature in the detected neutron pulse stream.

The delayed neutrons represent a characteristic signature of induced fission in the interrogated material and as such represent a key manifestation of the presence of SNM. The delayed neutron emission takes place when a β-decay nucleus (precursor) decays and the resulting daughter-nucleus emits a neutron. Delayed neutron emissions are commonly classified into six effective temporal groups (based on the contributory delayed neutron precursor half-lives and intensities) characterized by average half-lives between 0.2 and 56 s^44^. During the laser-driven active interrogation campaign, each laser-driven neutron pulse was followed by several minutes of data acquisition to allow sufficient time for all delayed neutron groups to fully decay and be recorded. Time-interval distributions of detected neutrons from the master and reference AWCC detectors were then constructed from the acquired data to evaluate the delayed neutron signature.

**Fig. 4:**
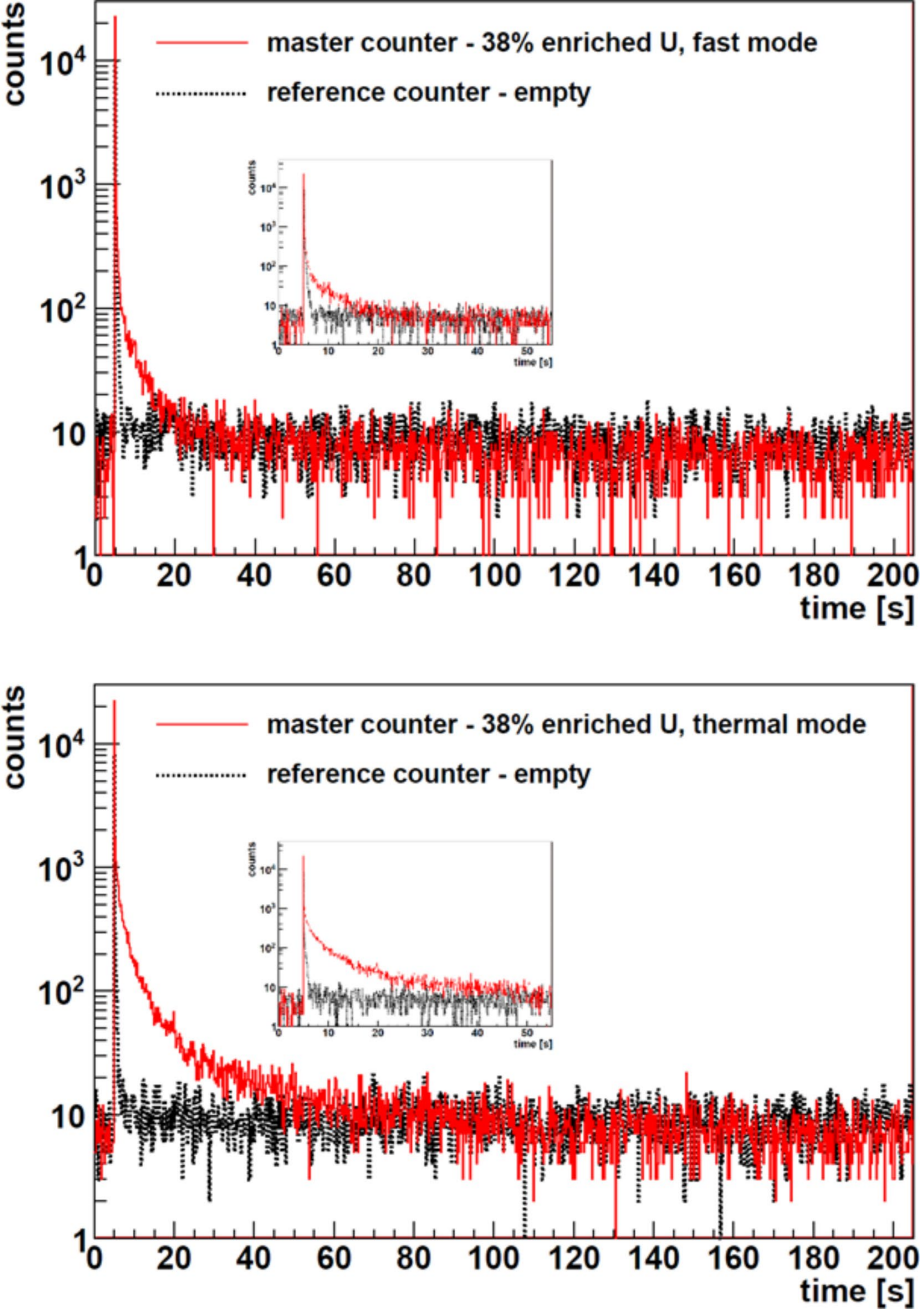
Time-interval distributions over 200 s following the laser trigger; (full red line) master AWCC containing 38% enriched U sample; (dotted black line) the empty, reference AWCC. AWCC detectors were operated in the ‘fast mode’ (top) and the ‘thermal mode’ (bottom). The insert highlights the detail of the time-interval distribution over 50 s following the trigger.

To illustrate the key features of the delayed neutron signature recorded in the AWCC detectors, the time distributions from laser-driven neutron interrogation of the 38% enriched uranium item are shown in Fig. 4 (the results for the 66% enriched uranium case are reported in Fig.A1 in the Supplementary Materials.) The distributions shown represent raw time distribution plots (not normalized to neutron yield) as recorded following two individual laser pulses with the two modes of AWCC operation – fast (Fig. 4 top) and thermal (Fig. 4 bottom). Red lines in the figure represent results from the master AWCC, while the time-interval distributions from the empty, reference AWCC are shown as black dotted lines. The sharp peak at the beginning of the time-interval distributions corresponds in time to the interrogating neutron pulse and is followed by a delayed component shown here on a timescale of 200 s. A net enhancement of the delayed neutron component in the time distributions is clearly visible compared to the empty reference AWCC. Insets in Fig. 4 provide a more detailed view of the delayed neutron signature over a timescale of 50 s.

As discussed in the previous section, the operation of the AWCC in the thermal mode offers greater sensitivity and so allows interrogation of smaller quantities of ^235^U (where self-shielding is reduced and thermal neutrons can interrogate the material effectively). In this configuration, the Cd liner of the inner wall of the central measurement well is removed, allowing for additional neutrons thermalized in the moderator to reach the item. This enhanced thermal neutron component also explains the increased delayed neutron signal observed in Fig. 4 bottom for the AWCC operated in the thermal mode for the same amount of interrogated SNM.

A closer look at the time-counting distributions from the reference AWCC, the one that contains no SNM and therefore no induced delayed fission neutrons, nevertheless reveals the presence of a delayed neutron component that decays away on significantly shorter timescales (order of 1 s) than the delayed signal observed in the other (master) AWCC that does contain uranium. This fast-decaying delayed signature was extensively studied in the previous laser-driven neutron production campaign^5^. It was identified as neutrons from the decay of ^9^Li produced in the Be converter under irradiation by high-energy deuterons from the target as well as via interactions with the subsequent high-energy produced neutrons. The ^9^Li production reactions are ^9^Be(d,2p)^9^Li and ^9^Be(n, p)^9^Li with thresholds of 18.4 MeV and 14.3 MeV, respectively, with the (d,2p) reaction being the dominant channel. ^9^Li is known to produce delayed neutrons with a half-life of 178.3 ms which matches the trace from the reference AWCC in Fig. 4. Importantly, the ^9^Li delayed neutron yield can serve as a laser-driven-ion diagnostic and this realization opens up a new avenue of research to support both experimental and theoretical developments. Distinct from this fast-decaying delayed component, the signal from the AWCC containing the SNM sample exhibits a delayed neutron signature over a much longer timescale consistent with the timescale of delayed fission neutrons. The delayed neutron emission was analyzed over a 200-second interval starting 1 s after the laser pulse. The 1-second delay was used to reduce the impact of the ^9^Li delayed neutron component since it corresponds to ~ 6× the ^9^Li half-life.

It should be emphasized that one of the key features of the LDNS based on the relativistic transparency acceleration mechanism is the production of a high-intensity, mostly forward-peaked, neutron pulse (> 10^10^ n/sr in this campaign), which has the potential to provide a statistically significant (high precision) delayed neutron signature in a single pulse. The time distributions presented illustrate this capability in an unambiguous way. This is the first demonstration of this new analytical capability.

The initial laser-target interaction is accompanied by an intense flash of X-rays^11^, and the potential contribution of high energy X-ray-induced fissions to the measured delayed neutron signature was evaluated. Photofission cross sections for ^235^U and ^238^U have an energy threshold of about 8 MeV, with a resonance behavior up to about 15 MeV. The cross-section peak value is, however, an order of magnitude less than the cross-section of neutron-induced fission in the same range of energy (10–20 MeV). A dedicated measurement was performed with 2 kg of depleted uranium (DU) material inside a high-level neutron coincidence counter version II (HLNCC-II) that is conceptually similar to an AWCC, but smaller and with less neutron efficiency^5,45^. The DU material was used bare and then subsequently shielded by a 10 cm thick Pb blocks to significantly reduce the (potential) contribution of X-rays to the induced fissions of depleted uranium without having a large effect on the transmitted fast-neutron neutron flux. The integral delayed neutron counts were normalized to bubble chambers located inside the HLNCC-II and were then evaluated for both DU configurations. The measured ratio of the bare versus shielded DU configuration corresponded to (1.00 ± 0.29), where the uncertainty is estimated as the 1-σ level. This suggests that any amount of fission caused by the X-rays is small (as expected). In order to improve the precision of the results a second experiment was performed with an intense laser-driven X-ray flash, obtained by substituting the CD-laser target with ^238^U disk targets (thickness 350 μm diameter 90 μm). The laser-driven electron distribution interacting with this thicker, high Z target gives an increase of magnitude in radiative losses (scaling with Z^2^ and density from CD to U) and so in the bremsstrahlung production of X-rays^46^. Such a flash was used to illuminate the 38% ^235^U enriched item, and the subsequent delayed neutron production was measured. The delayed neutrons production from this X-ray flash is only 2% of the DN production from a typical 30 mrem neutron shot. This result demonstrates the negligible effect of X-ray-induced fissions on the delayed neutron signature and confirms that neutron-induced fissions are the overwhelming source of the observed delayed neutron signal.

To quantify the performance of the interrogation method, the integral delayed neutron counts for the three high enriched uranium materials placed inside the master AWCC were evaluated. Note that the contribution of background neutrons was subtracted using the data recorded in the reference AWCC detector (the details of the procedure are described in the Methods section). Because of shot-to-shot variations in neutron intensity, the integral delayed neutron counts were normalized to the neutron dose measured in the bubble chambers located on the target chamber exit flange, directly on the neutron-beam axis. Bubble chambers with three sensitivities were used to ensure proper measurement dynamic range in every shot (see Methods). The measured neutron doses for the laser-driven neutron pulses reported in this paper corresponded to 5–37 mrem. An overview of the measured results is provided in Table 1. Note that some enriched uranium samples were interrogated with multiple neutron pulses to improve the statistical precision, and to monitor the reproducibility of the present test method. Table 1 shows results for all the individual short-pulse laser-driven neutron pulses where enriched uranium samples were present.

**Table 1:** Summary of normalized integral delayed neutron (DN) counts over a 1–200 s interval for three ^235^U enrichments studied in this work.

AWCC mode	^235^U enrichment (wt%)	Neutron dose at 0° target chamber exit flange (mrem)	Neutron dose uncertainty (mrem)	Normalized integral of DN counts (counts/mrem)	Uncertainty of normalized integral of DN counts (counts/mrem)
Fast	12	14.6	0.5	32	10
Fast	38	19.0	0.6	103	9
Fast	66	13.2	0.6	103	12
Fast	66	21.9	0.4	132	8
Thermal	12	5.0	0.2	601	40
Thermal	12	18.3	0.6	785	27
Thermal	12	28.7	0.9	695	22
Thermal	12	23.6	0.6	782	20
Thermal	38	13.6	0.4	953	30
Thermal	38	34.5	1.2	1039	38
Thermal	38	21.9	0.6	1059	30
Thermal	38	33.3	0.9	963	27
Thermal	38	36.9	0.6	854	16
Thermal	38	27.2	0.2	878	11
Thermal	66	20.6	0.6	1090	33
Thermal	66	11.2	0.3	1151	38
Thermal	66	9.1	0.3	911	35
Thermal	66	27.6	0.2	1085	11
Thermal	66	36.7	0.6	908	17
Thermal	66	27.6	0.2	1041	10
Thermal	66	23.0	0.4	1292	24
Thermal	66	17.1	0.2	1068	19
Thermal	66	30.4	0.8	1173	31

The results presented in Table 1 demonstrate that materials with all three enrichments evaluated in this work can be identified in a single laser-driven neutron pulse. Note that the uncertainties listed for the integral DN counts include the uncertainty on the bubble chamber readings used for normalization and also the uncertainty in the background corrected measured DN counts determined as the square root of the number of counts observed within the DN interval. A clear increasing trend of integral delayed neutron counts with ^235^U enrichment is observed for fast as well as thermal modes, as expected. In fast mode, the detection response is nearly linear, which makes particularly straightforward the determination of the amount of fissile material present in the canisters. The sensitivity of the active interrogation is significantly enhanced in the thermal mode, however the interrogation in thermal mode exhibits non-linear behavior for higher ^235^U enrichment of the items^47^ (see Fig. 4). At low enrichment, the ^235^U mass is more dilute in the sample, and as the ^235^U enrichment increases, the self-shielding effect reduces the thermal neutron penetration through the sample, so fast mode neutron interrogation is more representative.

As one approach, one could utilize a thermal mode high-sensitivity detector arrangement for high-throughput active interrogation for screening purposes. For the cases where fissile material is detected, one could set aside the item to repeat the assay offline safely with a fast mode detection arrangement (with increased neutron dose if necessary) in order to determine more accurately the amount of special nuclear material, and possibly to do additional tests as warranted (e.g., passive muon imaging, nuclear spectroscopy, etc.) before attempting internal inspection of the item.

## Discussion

A series of experiments were conducted at Los Alamos National Laboratory to explore the potential and technical advantages of active interrogation using a high-intensity, laser-driven neutron source. The results presented in this paper provide the first direct evidence of the feasibility of single-pulse laser-driven neutron interrogation of special nuclear materials through the detection of delayed neutrons. The reported data demonstrate the ability to detect the presence of fissile material unequivocally and with a level of consistency, which is necessary for practical quantitative applications. With the first successful integrated demonstration of active interrogation with LDNS to detect fissile material, this research advances the technology from a conceptual stage to a phase where validation has occurred in a laboratory environment. From this platform optimization and engineering studies can be initiated. Below, we suggest further work to further advance the technology out of the laboratory and towards the deployment of a production system.

### For further work towards a production system

In considering the future development and deployment of the LDNS active interrogation concept demonstrated in this study, it is pertinent to discuss the progression of its Technology Readiness Level (TRL). Specifically, the focus is on advancing to TRL 5, which involves “pre-prototype testing in a simulated environment in the laboratory.” To construct a pre-prototype, one must first define the specific mission to derive the necessary user requirements. A particular or representative application must be selected, contingent upon funding for research and development by a committed sponsor. This holds true not only for an LDNS but also for neutron sources driven by conventional accelerators, especially within the context of fissile material detection. For instance, the design of the detector system would vary significantly depending on whether the object under interrogation is the size of a suitcase, a radioactive-waste drum, or a semitrailer. Correspondingly, the required D-beam properties would be different as well.

Advancing to TRL 5 for most applications involving LDNS that we envision will necessitate the development of high-repetition-rate lasers and compatible target systems. This requirement is particularly critical for active interrogation applications that can demand high throughput and substantial neutron doses. The criteria to determine what constitutes ‘suitable’ equipment may differ among researchers and sponsors; nonetheless, we provide our brief evaluations here to inform and guide the broader enabling research in this field.

#### Laser targets

Our baseline concept for an LDNS is based on a laser-based D accelerator using the BOA mechanism based on the demonstrated level of performance and sufficient maturity. That requires sub-micron thick D-rich targets that can be replenished and repositioned accurately at the laser focal plane at a rate ranging from ~ 0.1–10 Hz, depending on specific application requirements. The present labor-intensive methodology for laboratory-based experiments involving mounting spin-coated plastic foils on a tiny holder that is manually mounted on the target holder in the chamber is clearly unsuitable. Promising techniques already demonstrated with high-power high-energy lasers are liquid crystals^48,49^ and liquid cryogenic D_2_ jets^50^. In the meantime, it is possible that alternative ion acceleration mechanisms such as collisionless-shock acceleration^14^ in thin deuterated foam targets ~ 10 μm thickness may demonstrate equivalent performance for this application. In that case, dedicated research and development may be warranted to develop a foam-target system compatible with high repetition-rate operations.

#### High repetition lasers

Although the CO_2_-based laser at the Brookhaven ATF merits continued attention, present state of the art performance in high-energy high-power short-pulse lasers and laser-driven ion acceleration is achieved with solid-state lasers, so we restrict our discussion to the latter. There is a clear need for transitioning away from general-purpose research laser facilities such as Trident, typically large in size and optimized for flexibility and access, towards more compact laser systems engineered and optimized with a view towards deployment in the field, with a much cheaper cost per shot and per J of laser energy. Improving the low repetition rate of lasers from the Trident generation is also important. While the technology of the low-energy high-repetition-rate front end on Trident was improved over time or even pioneered there (in the case of pulse cleaning), the low shot-repetition rate ~ 10^− 4^ Hz was dictated by the cooling time of the glass laser amplifiers, which were based on 1970’s laser technology. Much newer technology for relevant PW-class sub kJ laser pulses (per beamline) has already been demonstrated at repetition-rates up to 10 Hz, which is sufficient for most envisioned applications. While the exact laser system suitable for a TRL 5 demonstration is not quite available for purchase off-the-shelf, we believe that it could be delivered as a custom system under contract with a national laboratory or one of the large laser companies worldwide. We justify our assessment below and outline what such a laser might look like.

Two main relevant technology branches exist in high repetition-rate solid-state laser amplifiers: Ti: S crystals pumped by glass lasers and actively cooled glass disks. Ti: S amplifiers are the path to the highest powers and shortest pulse lengths (≈ 10–50 fs), limited to ~ 10 J per beamline at 800 nm wavelength by factors such as the availability of suitable crystals. They can operate at longer pulse lengths if required, which is a valuable feature for parametric studies in a multi-purpose facility. Intrinsically, they offer the tradeoff of higher intensity in shorter pulses in exchange for lower pulse energy and lower wall-plug efficiency, the latter in part because of the pumping step going from glass to Ti: S. On the other hand, state-of-the-art high repetition-rate glass disk amplifiers operate at 1 μm wavelength pumped by either flash lamps or diode arrays. They feature higher energy short-pulses per beam (> 100 J demonstrated at high repetition-rates) and higher energy efficiency (~ 10% if the diode is pumped). They are also intrinsically cheaper per J of delivered laser energy. However, they usually cannot support the bandwidth to go below sub-ps pulse duration, providing lower intensity for a given pulse energy. It is easy to see that both technologies have positive attributes, e.g., higher intensity is desirable (it may lead to a hotter D spectrum that exploits a higher nuclear-stripping cross-section in the neutron converter), but so is higher energy (it leads to more D ions). Therefore, the laser-pulse length is a key optimization parameter in LDNS-relevant D acceleration.

So far, the best ion acceleration results we have obtained have been based on BOA with sub-ps laser pulses. The ion dynamics of target-plasma disassembly that limit ion acceleration are ~ sub-ps. For a given laser-pulse energy, optimal target thickness decreases with shorter pulses^51^, which makes them more susceptible to destruction by pre-pulse, therefore increasing the pulse cleaning requirements possibly beyond the state of the art. A simple optimization for this regime (including laser cost) yields sub-ps optimum laser-pulse duration^21^. Consistent with this assessment, ion acceleration results from the highest energy Ti: S laser facilities have not matched those in glass-laser facilities. Therefore, while we strongly encourage continued research on ion acceleration with ~ fs laser pulses, at present glass lasers would be the right choice for reaching TRL 5 quickly with least risk. Within that branch, the application-specific requirements would determine whether ~ 1/minute repetition-rates (i.e., flash-lamp-pumped amplifiers) are sufficient or 10 Hz (i.e., diode-pumped amplifiers) are necessary.

We highlight two existing modern LDNS-relevant laser facilities that demonstrate the existence of sufficiently mature laser technology. Such lasers include the Penelope PW laser^52^ with diode-pumped glass amplifiers at the Helmholtz-Zentrum Dresden-Rossendorf (150 J in 0.15 ps at 1 Hz repetition-rate), and the L4 ATON4 laser beamline at ELI Beamlines (Prague)^53^ featuring flash-lamp-pumped glass amplifiers, which operates in 1 PW (150 J in 0.15 ps) and 10 PW (1.5 kJ in 0.15 ps) configurations at 1 shot/minute. As we explain below, a modern glass laser in the 1 PW class can be engineered to have a compact footprint suitable for deployment in the field to drive a LDNS. With similar pulse cleaning technology in the front end as the 200 TW Trident, it should be capable of driving a higher neutron dose/J by virtue of a higher possible intensity. But, even with the same efficiency, 1 PW should be adequate for most active interrogation applications we envision, likely with a single shot. Therefore, experiments at either L4 1 PW or Penelope should be strongly considered for further development of LDNS-based active interrogation, provided the demonstrated laser contrast is high enough and that regulatory hurdles relating to neutron generation and the interrogated sample can be overcome. Such a successful test at those facilities, if otherwise the rest of the hardware is a reasonably close prototype of a fieldable system, might be judged to be a TRL 5 demonstration.

A simplified version of the HAPLS laser^54^ (10 Hz repetition rate) built by LLNL for the L3 beamline at ELI Beamlines (Prague) is an attractive design basis for an LDNS prototype in a fieldable system. The footprint of the whole HAPLS laser is 4.6-by-17 m, plus 4 square meters for the laser pulse compressor, which represents an upper bound of what would be needed. HAPLS consists of two interconnected Livermore-designed laser systems: a diode-pumped, solid-state Nd: glass laser that pumps the second system, a Ti: S 30 fs, 30 J short-pulse laser. The pump laser is frequency doubled to deliver 200 J of energy at a repetition rate of 10 Hz. HAPLS has surpassed a much higher bar than would be needed for TRL 5. The design simplification to be considered is feeding a cleaned sub-ps pulse to a stretcher, then directly to the HAPLS glass pump amplifier, then finally to the pulse compressor back to sub-ps. Such a simplified design dispenses with all the unneeded fs-pulse and frequency-doubling hardware, the latter increasing the net amplifier energy output beyond 200 J, thus tripling the Trident pulse energy. So, in principle, a 1-second, 10-shot burst from such a modified-HAPLS laser driving a LDNS would have allowed us to perform the same measurement reported in this paper with at least 30x the neutron yield, probably more if the higher intensity capability (from higher pulse energy) were exploited.

#### Detections solutions

From the neutron detection perspective, potential solutions include traditional ^3^He-based systems but also alternatives such as scintillation detectors^32,34,36,37,40^. Scintillation detectors offer improved response times (both recovery and counting rate), a potential advantage with an LDNS pulse system. Excellent gamma-ray rejection will always be a prominent consideration. Recent developments in position-sensitive detectors based on ^6^Li scintillation composite technology provide spatial resolution for locating special nuclear material accurately^55^. Continued research and development will be crucial to realizing the full potential of LDNS-based active interrogation systems with detector selection depending on application specific goals, commercial and other considerations (reliability, stability, supply change, disposal costs etc.).

## Conclusions

The Los Alamos National Laboratory experiments reported here illustrate the potential of a high-intensity, laser-driven neutron source for active interrogation. The data obtained mark a significant milestone, providing the first empirical validation of single-pulse laser-driven neutron interrogation of special nuclear materials through delayed neutron detection. The ability to unequivocally detect the presence of fissile material and the consistency of the results demonstrate this technology’s practical feasibility.

This successful demonstration of active interrogation with LDNS signifies a progression of the technology from a theoretical concept to a validated laboratory proof-of-concept. It sets the stage for future advancements in this field, potentially revolutionizing the way we detect and handle fissile materials.

During the experimental campaign, as a result of several refinements, we achieved the highest LDNS yield yet reported, predominant forward-directed yield per solid angle, to drive the D-ion beam that, in turn, drives the neutron source. It is essential to emphasize the significance of these results for both the non-destructive assay and related experimental physics communities and theoretical plasma physics. The data presented in the paper offer an opportunity for theoretical physicists to validate and refine their plasma models and simulations, as well as for the designers of next-generation laser-driven neutron sources to develop platforms for practical applications.

This novel capability shows considerable promise for a wide range of applications in the realm of active interrogation, including high-throughput interrogation of transport containers at ports, treaty verification applications (nuclear warhead and nuclear material signatures), assay of radioactive debris, stockpile stewardship and certification, and spent nuclear fuel assay at storage facilities and in casks. The potential of LDNS for further applications and extensions to other areas of use is vast, including their use as alternatives with lower regulatory burden and cost than nuclear reactors or conventional accelerators for neutron production at universities. Areas such as basic material science research, industrial testing and in situ maintenance (e.g., jet turbine blades, bridge components) would benefit significantly from LDNS. Additionally, the high intensity and prevalent directionality of the laser-driven neutron pulse make it suitable for pulsed radiography of static and dynamic experiments^14,23,56^.

The technology and science of LDNS are currently progressing rapidly, and we anticipate the availability of mobile sources with automated target changers, optimized repetition rates, and tailored neutron energy spectra and directionality to meet the increasing safety, security, and safeguards needs.

## Methods

### Neutron counters

Both of the AWCCs used in the experiment were thoroughly characterized prior to commencement of the laser-driven interrogation measurements to assure comparable performance. Count rates of (103342 ± 47) cps and (102342 ± 39) cps were obtained in thermal mode for the same reference ^252^Cf spontaneous fission sealed neutron source in the master (assay) and reference (background) counters, respectively and confirmed better than 1% agreement in efficiency between the two detectors.

To obtain integral delayed neutron counts directly from the interrogated nuclear material a background neutron contribution had to be removed from the detected signal. This was performed using the data from the reference AWCC to account for the contribution of ^9^Li delayed neutron component. Note that in the very short times following the interrogating pulse (up to ~ 1s), the ambient neutron background is affected by a contribution of ^9^Li delayed neutrons produced through the interactions in the Be converter, and this contribution needs to be considered to ensure accurate background subtraction. The neutron background from the reference AWCC does not necessarily need to be equivalent to the background measured in the master AWCC with the nuclear material (slightly different location of each detector, differences in signal processing electronics). Therefore, the neutron background from the reference AWCC was scaled to match the AWCC used for nuclear material measurements. This was accomplished by extracting ambient background from the AWCC with nuclear material during the period immediately before the interrogating pulse. This period provides the best estimate of the ambient neutron background for this AWCC. The ambient neutron background from the reference AWCC was measured in the similar time period and the ratio of these two measurements provided the necessary scaling factor to correct for any residual differences between the reference AWCC and the AWCC with nuclear material. The signal from the reference AWCC for each shot is scaled by the appropriate scaling factor. The scaling factor was determined on a shot-by-shot basis.

### Neutron bubble detectors

Bubble detectors produced by Bubble Technology Industries (BTI)^57^ were used to measure the neutron dose. Multiple bubble detectors with three different bubble-to-dose sensitivities (in the range 5–19 bubble/mrem) were placed at 0° with respect to the laser propagation direction at the chamber’s exit flange. The neutron doses reported in the paper are the weighted average values of the detectors, and the uncertainty is the associated standard deviation of the values.

### Neutron yield determination

The neutron yield in neutron/sr is obtained as follows. The neutron dose in mrem was obtained from bubble detectors, and the neutron energy distribution was obtained from the nTOF measurements (Fig. 3). The neutron yield in neutron/cm^2^ at the bubble detector location is calculated by multiplying neutron dose for the integral, in the neutron energy range, of the ratio between the normalized energy spectrum and the neutron sensitivity^11,57,58^, we then convert the yield to neutron/sr by knowing the distance of the bubble detector to the neutron source (104 cm for the exit flange on axis)^12^. Bubble detector neutron sensitivity data from the cited literature were linearly interpolated to be used in the calculation.

### Neutron time-of-flight (nTOF)

A neutron time-of-flight (nTOF) detector system, based on a NE102 plastic scintillator (76.2 mm diameter, 18.2 mm thick) coupled to XP4362B with 6 dynodes Photonis PMT was positioned 6.2 m away from the Be converter along the central beam axis (the laser-propagation direction and the symmetry axis of the converter), outside the Trident building. Positioning the detector outside the building reduced the neutron scattering contributions into the scintillation detector significantly. The nTOF was operated in current mode and was connected to a fast oscilloscope. The nTOF spectrum consists of a digitized oscilloscope trace of the photomultiplier output. 102 mm of lead was placed in front of the detector to attenuate the X-ray flash associated with the pulse. A pure X-ray shot was produced in Trident, using a ^238^U thin target instead of the CD target, to acquire the X-ray response function of the neutron detector system for subtraction to obtain a neutron–only spectrum. The neutron energy spectrum was then unfolded by fitting the data with response vectors calculated using the MCNP-Polimi radiation transport code (using ENDF VII cross-section libraries)^59^. The response vectors were matched to the 20-ns nTOF bins in the experimental spectrum. The nTOF spectrum was described as a linear combination of overlapping response functions. All the recoil and reaction contributions in the scintillation material (proton, alpha, carbon) and the corresponding light output contributions to the signal were calculated as needed for the neutron response function of the detector.

## Electronic supplementary material

Below is the link to the electronic supplementary material.

Supplementary Material 1

## Data Availability

Data are provided within the manuscript or supplementary information files.
