# Supplementary material for: Demonstration of active neutron interrogation of special nuclear materials using a high-intensity short-pulse-laser-driven neutron source
Source: Sci Rep. 2025 Jan 3;15:724. doi: 10.1038/s41598-024-82641-y (PMC11699058; doi:10.1038/s41598-024-82641-y)
Supplement: Supplementary file 1 — Supplementary Material 1 [file 41598_2024_82641_MOESM1_ESM.docx]

Supplementary material to

“Demonstration of Active Neutron Interrogation of Special Nuclear Materials Using a High-Intensity Short-Pulse-Laser-Driven Neutron Source”

Case: 66% U enrichment, fast and thermal mode interrogation
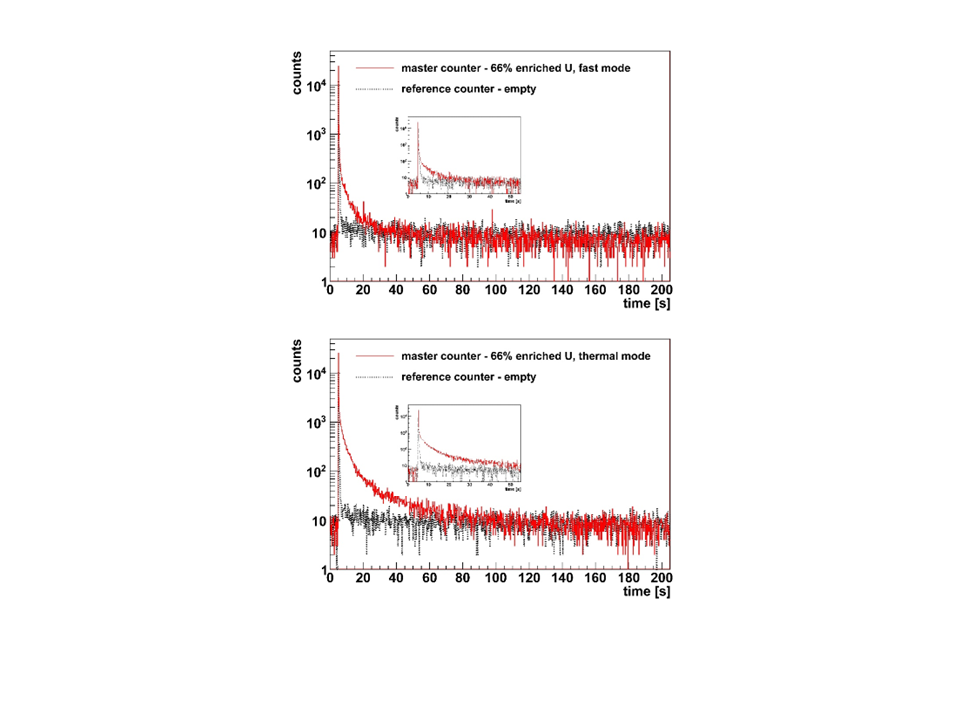


**Figure A1:** Time-interval distributions over 200 s following the laser trigger; (full red line) master AWCC containing 66% enriched U sample; (dotted black line) the empty, reference AWCC. AWCC detectors were operated in the ‘fast mode’ (top) and the ‘thermal mode’ (bottom). The insert highlights the detail of the time-interval distribution over 50 s following the trigger.
